# Supplementary material for: The Systems Biology Research Tool: evolvable open-source software
Source: BMC Syst Biol. 2008 Jun 29;2:55. doi: 10.1186/1752-0509-2-55 (PMC2446383; doi:10.1186/1752-0509-2-55)
Supplement: Additional file 1 — SBRT Archive. An archive of the current version of the Systems Biology Research Tool. [file 1752-0509-2-55-S1.zip › sbrt-1.4.0/doc/developers_guide/api/sbrt/kernel/math/HashLinearComb.html]

HashLinearComb


|  |  |  |
| --- | --- | --- |
| **Overview** \| **Package** \| **Class** \| **Use** \| **Tree** \| **Deprecated** \| **Index** \| **Help** | |  |
| **PREV CLASS**   **NEXT CLASS** | **FRAMES**    **NO FRAMES**     **All Classes** |  |
| SUMMARY: NESTED \| FIELD \| CONSTR \| METHOD | DETAIL: FIELD \| CONSTR \| METHOD |  |


---


## sbrt.kernel.math Class HashLinearComb<V>

```
java.lang.Object
  sbrt.kernel.math.AbstractPolynomial<V>
      sbrt.kernel.math.AbstractLinearComb<V>
          sbrt.kernel.math.HashLinearComb<V>
```

**Type Parameters:**: `V` - the variable type.

**All Implemented Interfaces:**: java.lang.Iterable<LinearComb.Term<V>>, LinearComb<V>, MathExpr<V>, Polynomial<V>

---

``` public final class HashLinearComb<V> extends AbstractLinearComb<V> ```

This class is a concrete implemenation of `LinearComb`.
The hash codes of the provided variables are used extenstively in this
implementation to provide constant-time lookup for internally stored
information.

**Author:**
:   This class was written and documented by Jeremiah Wright
    while in the Wagner lab.

---

| **Nested Class Summary** | |
| --- | --- |
| `static class` | `HashLinearComb.Term<V>`             This class is a concrete implementation of `LinearComb.Term`. |


**Constructor Summary**

`HashLinearComb(java.util.Collection<? extends LinearComb.Term<? extends V>> terms,
double constant,
double zeroCutoff)`
  
          Constructs a new linear combination from the provided terms and
constant.

`HashLinearComb(LinearComb.Term<? extends V>[] terms,
double constant,
double zeroCutoff)`
  
          Constructs a new linear combination from the provided terms and
constant.


| **Method Summary** | |
| --- | --- |
| `HashLinearComb<V>` | `add(double constant)`             Adds, in the mathematical sense, the provided constant to this linear combination. |
| `HashLinearComb<V>` | `add(LinearComb<? extends V> expression)`             Adds, in the mathematical sense, the provided linear combination to this linear combination. |
| `double` | `getCoefficient(java.lang.Object variable)`             Returns the coefficient of the term containing the provided variable. |
| `HashLinearComb.Term<V>[]` | `getTermArray()`             Returns the terms of this linear combination as an array. |
| `java.util.Set<V>` | `getVariables()`             Returns the set of variables contained in this mathematical expression. |
| `boolean` | `hasAllVariables(java.util.Collection<?> variables)`             Indicates if this mathematical expression contains all of the variables in the provided collection. |
| `boolean` | `hasVariable(java.lang.Object variable)`             Indicates if the provided object is a variable contained in this mathematical expression. |
| `boolean` | `hasVariables()`             Indicates if this mathematical expression contains any variables. |
| `java.util.Iterator<LinearComb.Term<V>>` | `iterator()`             Returns an iterator over the terms of this linear combination. |
| `static void` | `main(java.lang.String[] args)`             Used for testing purposes. |
| `HashLinearComb<V>` | `multiply(double constant)`             Multiplies this linear combination by the provided constant. |
| `HashLinearComb<V>` | `plugIn(java.util.Map<?,? extends LinearComb<? extends V>> variableValues)`             Plugs in the provided linear combinations to produce a new linear combination. |
| `HashLinearComb<V>` | `plugIn(java.util.Map<?,java.lang.Double> numbers, java.util.Map<?,? extends LinearComb<? extends V>> linearCombs)`             Plugs in the provided numerical values and linear combinations to produce a new linear combination. |
| `HashLinearComb<V>` | `plugIn(java.lang.Object variable, double value)`             Plugs the provided numerical value into the provided variable to produce a new linear combination. |
| `HashLinearComb<V>` | `plugIn(java.lang.Object variable, LinearComb<? extends V> expr)`             Plugs the provided linear combination into the provided variable to produce a new linear combination. |
| `HashLinearComb<V>` | `plugInNumbers(java.util.Map<?,java.lang.Double> variableValues)`             Plugs in the provided numerical values to produce a new linear combination. |
| `static <V> HashLinearComb<V>` | `singleTerm(double coefficient, V variable)`             Returns a single-term linear combination with the provided variable and coefficient. |
| `static <V> HashLinearComb<V>` | `singleTerm(LinearComb.Term<V> term)`             Returns a single-term linear combination with the provided term. |
| `static <V> HashLinearComb<V>` | `singleTerm(V variable)`             Returns a single-term linear combination with the provided variable. |
| `static <V> HashLinearComb<V>` | `singleTerm(V variable, double coefficient)`             Returns a single-term linear combination with the provided variable and coefficient. |
| `static <V> HashLinearComb<V>` | `singleTerm(V variable, double coefficient, double zeroCutoff)`             Returns a single-term linear combination with the provided variable, coefficient, and zero cutoff. |
| `HashLinearComb<V>` | `solveFor(java.lang.Object variable, double rightHandSide)`             Equates this linear combination to the provided constant and solves for the provided variable. |
| `int` | `terms()`             Returns the number of terms contained in this linear combination. |
| `int` | `variables()`             Returns the number of variables in this mathematical expression. |

**Methods inherited from class sbrt.kernel.math.AbstractLinearComb**

`equals, getTermList, getZeroCutoff`

**Methods inherited from class sbrt.kernel.math.AbstractPolynomial**

`checkConstant, getConstant, hashCode, toString`

**Methods inherited from class java.lang.Object**

`clone, finalize, getClass, notify, notifyAll, wait, wait, wait`

**Methods inherited from interface sbrt.kernel.math.LinearComb**

`hashCode, toString`

**Methods inherited from interface sbrt.kernel.math.Polynomial**

`getConstant`

**Constructor Detail**

### HashLinearComb

```
public HashLinearComb(LinearComb.Term<? extends V>[] terms,
                      double constant,
                      double zeroCutoff)
```

:   Constructs a new linear combination from the provided terms and
    constant. If the absolute value of coefficient of a term in the
    provided array is less than the provided zero cutoff, that term
    will not be included in the new linear combination.

    **Parameters:**: `terms` - an array containing the terms used to create this linear combination.: `constant` - the constant of this linear combination.: `zeroCutoff` - the zero cutoff.

---


### HashLinearComb

```
public HashLinearComb(java.util.Collection<? extends LinearComb.Term<? extends V>> terms,
                      double constant,
                      double zeroCutoff)
```

:   Constructs a new linear combination from the provided terms and
    constant. If the absolute value of coefficient of a term in the
    provided collection is less than the provided zero cutoff, that term
    will not be included in the new linear combination.

    **Parameters:**: `terms` - a collection of terms used to create this linear combination.: `constant` - the constant of this linear combination.: `zeroCutoff` - the zero cutoff. **Throws:**: `java.lang.IllegalArgumentException` - if a variable occurs in more than one provided term.: `java.lang.IllegalArgumentException` - if a provided value is either infinite or NaN; or if the zero cutoff is negative.


**Method Detail**

### getTermArray

```
public HashLinearComb.Term<V>[] getTermArray()
```

:   Returns the terms of this linear combination as an array.

    :   **Returns:**: the terms of this linear combination as an array.

---


### terms

```
public int terms()
```

:   Returns the number of terms contained in this linear combination.
    The numerical constant is not considered a term.

    :   **Returns:**: the number of terms contained in this linear combination.

---


### plugIn

```
public HashLinearComb<V> plugIn(java.util.Map<?,? extends LinearComb<? extends V>> variableValues)
```

:   Plugs in the provided linear combinations to produce a new
    linear combination.

    If some of the keys in the provided map are not variables
    in this expression, their provided value is simply ignored.

    :   **Parameters:**: `variableValues` - objects mapped to linear combinations. **Returns:**: the linear combination obtained by "plugging in" the provided linear combinations.

---


### plugIn

```
public HashLinearComb<V> plugIn(java.util.Map<?,java.lang.Double> numbers,
                                java.util.Map<?,? extends LinearComb<? extends V>> linearCombs)
```

:   Plugs in the provided numerical values and linear combinations to
    produce a new linear combination.

    If some of the keys in either of the provided maps are not variables
    in this expression, their provided values are simply ignored.

    :   **Parameters:**: `numbers` - objects mapped to numerical values.: `linearCombs` - objects mapped to linear combinations. **Returns:**: the linear combination obtained by "plugging in" the provided numerical values and linear combinations. **See Also:**: `plugIn(Map)`, `plugInNumbers(Map)`

---


### plugInNumbers

```
public HashLinearComb<V> plugInNumbers(java.util.Map<?,java.lang.Double> variableValues)
```

:   Plugs in the provided numerical values to produce a new
    linear combination.

    If some of the keys in the provided map are not variables
    in this expression, their provided value is simply ignored.
    If a value is provided for each variable in this linear
    combination, the resulting expression will not contain any
    variables of its own, only a constant.

    :   **Parameters:**: `variableValues` - objects mapped to numerical values. **Returns:**: the linear combination obtained by "plugging in" the provided numerical values.

---


### plugIn

```
public HashLinearComb<V> plugIn(java.lang.Object variable,
                                double value)
```

:   Plugs the provided numerical value into the provided variable to
    produce a new linear combination.

    :   **Parameters:**: `variable` - the variable whose value is to be assigned.: `value` - the value to be assigned to the provided variable in this linear combination. **Returns:**: the linear combination obtained by "plugging in" the provided numerical value; or this linear combination if the provided object is not a variable.

---


### plugIn

```
public HashLinearComb<V> plugIn(java.lang.Object variable,
                                LinearComb<? extends V> expr)
```

:   Plugs the provided linear combination into the provided variable to
    produce a new linear combination.

    :   **Parameters:**: `variable` - the variable whose value is to be assigned.: `expr` - the value to be assigned to the provided variable in this linear combination. **Returns:**: the linear combination obtained by "plugging in" the provided linear combination; or this linear combination if the provided object is not a variable.

---


### getCoefficient

```
public double getCoefficient(java.lang.Object variable)
```

:   Returns the coefficient of the term containing the provided variable.
    If the provided variable does not exist in this linear combination,
    it's coefficient equals 0.

    :   **Parameters:**: `variable` - the variable whose corresponding coefficient is to be returned. **Returns:**: the coefficient of the term containing the provided variable.

---


### solveFor

```
public HashLinearComb<V> solveFor(java.lang.Object variable,
                                  double rightHandSide)
```

:   Equates this linear combination to the provided constant and solves for
    the provided variable.

    :   **Parameters:**: `variable` - the variable in this linear combination for which to solve.: `rightHandSide` - the constant with which this linear combination is equated. **Returns:**: a new linear combination representing the value of the provided variable.

---


### add

```
public HashLinearComb<V> add(double constant)
```

:   Adds, in the mathematical sense, the provided constant to this
    linear combination.

    :   **Parameters:**: `constant` - the constant with which to add. **Returns:**: the result of adding this linear combination with the provided constant.

---


### add

```
public HashLinearComb<V> add(LinearComb<? extends V> expression)
```

:   Adds, in the mathematical sense, the provided linear combination to this
    linear combination.

    :   **Parameters:**: `expression` - the linear combination with which to add. **Returns:**: the result of adding this linear combination with the one provided.

---


### multiply

```
public HashLinearComb<V> multiply(double constant)
```

:   Multiplies this linear combination by the provided constant.

    :   **Parameters:**: `constant` - the constant with which to multiply this linear combination. **Returns:**: the result of multiplying this linear combination by the provided constant.

---


### getVariables

```
public java.util.Set<V> getVariables()
```

:   Returns the set of variables contained in this
    mathematical expression.

    :   **Returns:**: the set of variables contained in this mathematical expression.

---


### hasVariables

```
public boolean hasVariables()
```

:   Indicates if this mathematical expression contains any
    variables.

    :   **Returns:**: `true` if this expression contains at least one variable; `false` otherwise.

---


### hasAllVariables

```
public boolean hasAllVariables(java.util.Collection<?> variables)
```

:   Indicates if this mathematical expression contains all
    of the variables in the provided collection.

    :   **Parameters:**: `variables` - the collection of variables whose presence in this expression is to be tested. **Returns:**: `true` if each element of the provided collection is a variable in this expression; `false` otherwise.

---


### hasVariable

```
public boolean hasVariable(java.lang.Object variable)
```

:   Indicates if the provided object is a variable contained
    in this mathematical expression.

    :   **Parameters:**: `variable` - the variable whose presence in this mathematical expression is to be tested. **Returns:**: `true` if this expression contains the provided variable; `false` otherwise.

---


### variables

```
public int variables()
```

:   Returns the number of variables in this mathematical
    expression.

    :   **Returns:**: the number of variables in this mathematical expression.

---


### iterator

```
public java.util.Iterator<LinearComb.Term<V>> iterator()
```

:   Returns an iterator over the terms of this linear combination.

    :   **Specified by:**: `iterator` in interface `java.lang.Iterable<LinearComb.Term<V>>` **Specified by:**: `iterator` in interface `LinearComb<V>` **Overrides:**: `iterator` in class `AbstractLinearComb<V>`
    :   **Returns:**: an iterator over the terms of this linear combination.

---


### singleTerm

```
public static <V> HashLinearComb<V> singleTerm(V variable,
                                               double coefficient)
```

:   Returns a single-term linear combination with the provided variable and
    coefficient. The zero cutoff is set to 0.

    :   **Type Parameters:**: `V` - the variable type. **Parameters:**: `variable` - the variable.: `coefficient` - the coefficient. **Returns:**: a linear combination containing a single term with the provided variable and constant.

---


### singleTerm

```
public static <V> HashLinearComb<V> singleTerm(V variable,
                                               double coefficient,
                                               double zeroCutoff)
```

:   Returns a single-term linear combination with the provided variable,
    coefficient, and zero cutoff.

    :   **Type Parameters:**: `V` - the variable type. **Parameters:**: `variable` - the variable.: `coefficient` - the coefficient.: `zeroCutoff` - the zero cutoff. **Returns:**: a linear combination containing a single term with the provided variable, constant, and zero cutoff.

---


### singleTerm

```
public static <V> HashLinearComb<V> singleTerm(double coefficient,
                                               V variable)
```

:   Returns a single-term linear combination with the provided variable and
    coefficient. The zero cutoff is set to 0.

    :   **Type Parameters:**: `V` - the variable type. **Parameters:**: `coefficient` - the coefficient.: `variable` - the variable. **Returns:**: a linear combination containing a single term with the provided variable and constant.

---


### singleTerm

```
public static <V> HashLinearComb<V> singleTerm(V variable)
```

:   Returns a single-term linear combination with the provided variable.
    The coefficient of the term containing this variable is 1, and the
    zero cutoff is 0.

    :   **Type Parameters:**: `V` - the variable type. **Parameters:**: `variable` - the variable. **Returns:**: a linear combination containing a single term with the provided variable.

---


### singleTerm

```
public static <V> HashLinearComb<V> singleTerm(LinearComb.Term<V> term)
```

:   Returns a single-term linear combination with the provided term.
    The zero cutoff is set to 0.

    :   **Type Parameters:**: `V` - the variable type. **Parameters:**: `term` - the term. **Returns:**: a linear combination containing a single term with the provided variable.

---


### main

```
public static void main(java.lang.String[] args)
```

:   Used for testing purposes.

    :   **Parameters:**: `args` - none required.


---


|  |  |  |
| --- | --- | --- |
| **Overview** \| **Package** \| **Class** \| **Use** \| **Tree** \| **Deprecated** \| **Index** \| **Help** | |  |
| **PREV CLASS**   **NEXT CLASS** | **FRAMES**    **NO FRAMES**     **All Classes** |  |
| SUMMARY: NESTED \| FIELD \| CONSTR \| METHOD | DETAIL: FIELD \| CONSTR \| METHOD |  |


---
